# Mutual independence of alkaline‐ and calcium‐mediated signalling in *Aspergillus fumigatus* refutes the existence of a conserved druggable signalling nexus

**DOI:** 10.1111/mmi.13840

**Published:** 2017-11-14

**Authors:** Omar Loss, Margherita Bertuzzi, Yu Yan, Natalie Fedorova, Bethany L. McCann, Darius Armstrong‐James, Eduardo A. Espeso, Nick D. Read, William C. Nierman, Elaine M. Bignell

**Affiliations:** ^1^ Microbiology Section, Centre for Molecular Microbiology and Infection, Imperial College London London SW7 2AZ UK; ^2^ Manchester Fungal Infection Group, Division of Infection, Immunity and Respiratory Medicine University of Manchester Manchester M13 9NT UK; ^3^ The J. Craig Venter Institute, Infectious Diseases Program Rockville MD USA; ^4^ Fungal Pathogens Laboratory National Heart and Lung Institute, Imperial College London SW7 2AY UK; ^5^ Department of Molecular and Cellular Biology Centro de Investigaciones Biologicas (C.S.I.C.) Madrid Spain; ^6^ Present address: Ipsen Bioinnovation Ltd Abingdon UK

## Abstract

Functional coupling of calcium‐ and alkaline responsive signalling occurs in multiple fungi to afford efficient cation homeostasis. Host microenvironments exert alkaline stress and potentially toxic concentrations of Ca^2+^, such that highly conserved regulators of both calcium‐ (Crz) and pH‐ (PacC/Rim101) responsive signalling are crucial for fungal pathogenicity. Drugs targeting calcineurin are potent antifungal agents but also perturb human immunity thereby negating their use as anti‐infectives, abrogation of alkaline signalling has, therefore, been postulated as an adjunctive antifungal strategy. We examined the interdependency of pH‐ and calcium‐mediated signalling in *Aspergillus fumigatus* and found that calcium chelation severely impedes hyphal growth indicating a critical requirement for this ion independently of ambient pH. Transcriptomic responses to alkaline pH or calcium excess exhibited minimal similarity. Mutants lacking calcineurin, or its client CrzA, displayed normal alkaline tolerance and nuclear translocation of CrzA was unaffected by ambient pH. Expression of a highly conserved, alkaline‐regulated, sodium ATPase was tolerant of genetic or chemical perturbations of calcium‐mediated signalling, but abolished in null mutants of the pH‐responsive transcription factor PacC, and PacC proteolytic processing occurred normally during calcium excess. Taken together our data demonstrate that in *A*. *fumigatus* the regulatory hierarchy governing alkaline tolerance circumvents calcineurin signalling.

## Introduction

To successfully colonise the human host, pathogenic fungi must appropriately integrate multiple sensory, signalling and adaptation events under hostile environmental conditions (Grice *et al*., 2013). From the perspective of novel broad‐spectrum antifungal therapies the conserved nodes which govern hierarchical and co‐ordinated regulation of such adaptation is of great interest, and synergy of genetic and therapeutic perturbations can provide valuable insights into pharmacological modes of action, as well as informing the rationalised design of combination therapies.

The antifungal activities of multiple classes of calcium‐active drugs has been well‐documented (Afeltra and Verweij, 2003; Courchesne and Ozturk, 2003) suggesting a vital role for calcium homeostasis in fungal cells. However, the drugs which most potently target fungal calcium signalling also act upon mammalian cells, usually to lessen the efficacy of immune responses. Examples include the immunosuppressors FK506 and cyclosporine A, both of which, via binding to the peptidyl prolyl isomerases FKBP and cyclophilin, respectively, inhibit fungal calcineurin (Liu *et al*., 1991). One way to overcome this problem would be to concomitantly target co‐operatively acting fungal signalling pathways such that lower dosing of calcineurin‐active drugs could be used in combination with other agents.

Calcium is essential for the growth, stress tolerance and differentiation of pathogenic fungi (Jackson and Heath, 1989; Jackson and Heath, 1993; Silverman‐Gavrila and Lew, 2000). Paradoxically, however, the host environment contains a potentially lethal concentration (∼ 2 mM) of calcium ions (Blankenship *et al*., 2003; Blankenship and Heitman, 2005). Calcium signalling requires regulated influx of extracellular calcium which activates calcineurin‐mediated signalling and Crz‐mediated transcriptional responses to maintain cytosolic free calcium at a very low (50–100 nM) resting levels (Cunningham and Fink, 1994). Accordingly, mutation of the highly conserved calcium‐dependent protein phosphatase calcineurin, which regulates multiple aspects of cellular physiology and morphogenesis, severely impacts fungal growth, differentiation and virulence in multiple fungal pathogens (Odom *et al*., 1997; Blankenship *et al*., 2003; Sanglard *et al*., 2003; Blankenship and Heitman, 2005; Steinbach *et al*., 2006).

Fungal calcineurin is a heterodimeric protein consisting of catalytic and Ca^2+^/calmodulin binding units. In the major pathogenic mould *Aspergillus fumigatus*, null mutants of the CalA/CnaA catalytic subunit show severe defects in hyphal growth and virulence (Steinbach *et al*., 2006; da Silva Ferreira *et al*., 2007). In the encapsulated yeast pathogen *Cryptococcus neoformans* the *CNA1* gene product is required for *in vitro* survival under physiological conditions mimicking those found in the host (37°C, alkaline pH, 5% CO_2_), and for virulence in rabbits (Odom *et al*., 1997). In *Candida albicans*, calcineurin is not required for the yeast to hyphal transition, host cell adherence or injury, but is required for tolerance of alkaline and ionic stresses (Bader *et al*., 2003) and protects the fungus from the stress caused by endogenous levels of calcium in serum. Thus *C*. *albicans* calcineurin acts in a homeostatic sense to permit passage through the bloodstream, a prerequisite for establishment of deep‐seated infections (Bader *et al*., 2003; Blankenship *et al*., 2003). An important subset of fungal calcineurin‐dependent functions are governed by the Crz family of calcium‐responsive transcription factors, whose subcellular localisation is dictated by phosphorylation status. In response to stimuli which promote elevation of cytosolic calcium, Crz transcription factors are dephosphorylated by calcineurin and transported into the nucleus of the cell. Fungal Crz transcription factors govern ion homeostasis, most notably that of calcium ions (Yoshimoto *et al*., 2002; Karababa *et al*., 2006).

In fungi, sensing and adaptation to alkaline stress is also governed by the highly conserved PacC/Rim101p family of transcription factors (Caddick *et al*., 1986; Tilburn *et al*., 1995; Li and Mitchell, 1997; Davis *et al*., 2000a, 2000b). Proteolytic processing and activation of the PacC/Rim101p transcription factors requiring six upstream Pal/Rim signalling proteins allows fungal species to withstand pH fluctuations, including those encountered within the mammalian niche. Accordingly, PacC/Rim101p signalling has been demonstrated to have a crucial role in the pathogenicity of *C*. *albicans* (Davis *et al*., 2000a, 2000b; Nobile *et al*., 2008), *A*. *nidulans* (Bignell *et al*., 2005) and *A*. *fumigatus* (Bertuzzi *et al*., 2014).

The functional coupling of calcium‐mediated signalling and alkaline tolerance has, in fungi, been evidenced on multiple levels including, in *S*. *cerevisiae*, by quantification of cytosolic calcium (Viladevall *et al*., 2004), intersection of alkaline‐responsive and calcineurin‐ and Crz1p‐dependent gene expression (Serrano *et al*., 2002; Viladevall *et al*., 2004; Roque *et al*., 2016), alkaline‐induced nuclear translocation of Crz1p (Ruiz *et al*., 2008) and through phenotypic analyses of pH‐ and FK506‐sensitive mutants (Viladevall *et al*., 2004). In *A*. *nidulans* and *C*. *neoformans*, mutants defective in calcineurin‐mediated signalling are intolerant of alkaline stress (Odom *et al*., 1997; Bader *et al*., 2003; Spielvogel *et al*., 2008), and in *C*. *albicans* integration of calcineurin/Crz1p‐ and Rim101p‐mediated signalling is required for alkaline tolerance (Kullas *et al*., 2007) and expression of alkaline‐responsive genes (Wang *et al*., 2011). The mechanistic basis of such functional coupling lies with alkaline‐induction of highly localised subcellular calcium transients prompting, in *S*. *cerevisiae* and *C*. *albicans*, the activation of calcineurin‐mediated Crz1p signalling (Viladevall *et al*., 2004; Wang *et al*., 2011). Crz1p‐mediated signalling occurs simultaneously, and in parallel, with Rim101p activation at alkaline pH and convergence of the respective functional outputs can be explained by commonality of target gene promoters, for example that of Sc*ENA1*, which is subject to regulation by a minimum of five different transcription factors (Ruiz and Arino, 2007). In a previous study, we demonstrated, by comparative transcriptomic analyses, significant concordance between *A*. *fumigatus* genes which are preferentially expressed during adaptation to the mammalian host niche, and those which are upregulated in response to alkaline stress *in vitro* (McDonagh *et al*., 2008). Among them were included two homologues of the *S*. *cerevisiae* vacuolar calcium ATPase *PMC1*, which modulates cytoplasmic calcium levels, as well as genes known to have a calcineurin‐dependent role in alkaline pH adaptation in yeast, such as *ENA1* and *PHO89*. This suggested a possible co‐operation between alkaline and calcium signalling during *A*. *fumigatus* infections. Given the suggested therapeutic relevance of coupled pH‐responsive and calcium‐responsive signalling in pathogenic fungi, and increasing evidence that membrane determinants of PacC/Rim101p‐mediated signalling will be druggable (Bignell, 2012; Cornet and Gaillardin, 2014; Lucena‐Agell *et al*., 2016), we sought to determine whether the highly conserved CrzA‐ and PacC‐mediated calcium and alkaline signalling mechanisms in *A*. *fumigatus* collaborate to effect alkaline tolerance.

## Results

### Role of extracellular calcium in *A. fumigatus* growth and alkaline tolerance

**Figure 1:**
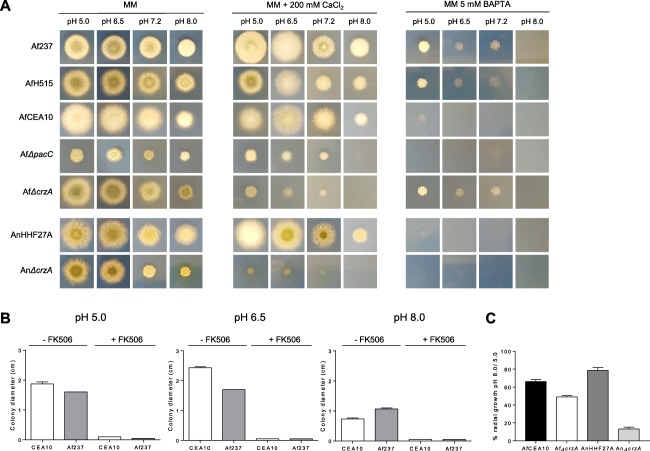
Requirement for extracellular calcium during *A*. *fumigatus* growth. A. Colonial growth of *A*. *fumigatus* isolates in the presence (MM), absence (5 mM BAPTA) or excess (200 mM CaCl_2_) of extracellular calcium ions, across a range of pH values. Colonial growth of the CrzA null mutant is drastically reduced in the presence of 200 mM CaCl_2_. B. Abrogation of calcineurin‐mediated signalling using FK506 (500 nM) drastically impairs mycelial outgrow from fungal colonies in a pH‐independent manner. C. Radial growth across a range of pH values of a wild type clinical isolate (CEA10) and isogenic CrzA null mutant and *A*. *nidulans* wild type (HHF27A) and isogenic CrzA null mutant (An*ΔcrzA*, HHF27L). Error bars show the Standard Error of the Mean (SEM).

The precise requirement for extracellular calcium during *A*. *fumigatus* growth and alkaline stress adaption has not been previously tested. We found that *A*. *fumigatus* clinical isolates, and derivatives thereof, can withstand high extracellular calcium concentrations without suffering adverse effects (0.2 M and greater, data not shown) and that growth is well supported by trace amounts of calcium ions supplied in standard, calcium nonsupplemented minimal medium (MM at pH 6.5). To test the requirement for extracellular calcium during *A*. *fumigatus* growth and pH‐adaptation we chelated trace calcium ions in a range of pH‐buffered growth media using the membrane‐impermeable calcium chelator BAPTA (Fig. 1A). Acquisition of extracellular calcium was found to be essential for growth at all tested pHs since addition of 5 mM BAPTA drastically impaired fungal growth. In order to exclude BAPTA‐mediated toxicity as the cause of growth retardation, we supplemented growth of the fungus with 200 mM CaCl_2_ in the presence of 5 mM BAPTA and were able to rescue growth at all tested pHs (Supporting Information Fig. S1). *A*. *fumigatus* colonial growth and sporulation are evident at all pHs tested, but relative to neutral pHs (pH 6.5–7.0) colonial growth is more compact at pH 8.0 with minimal outgrowth of hyphae into the surrounding agar (Fig. 1A and B).

If *A*. *fumigatus* calcineurin signalling is important for alkaline tolerance the antifungal potency of drugs inhibiting calcineurin activity would be expected to heighten at alkaline pH. In order to test this hypothesis we assessed the growth of *A*. *fumigatus* on pH‐buffered minimal medium containing a growth subinhibitory concentration of the calcineurin inhibitor FK506 (Fig. 1B). The presence of 1 mM (1 µg/ml) FK506 severely impeded the growth of all isolates tested. Following growth for 48 h in the presence of this agent, fungal colonies were visible but were extremely small relative to growth in the absence of FK506. No impact upon colony morphology or radial growth was observed at either high or low pH values suggesting that alkaline tolerance is not dependent upon calcineurin‐mediated calcium signalling in *A*. *fumigatus*. In further support of this conclusion, an *A*. *fumigatus* mutant lacking the calcineurin‐dependent transcription factor CrzA (Soriani *et al*., 2008; Soriani *et al*., 2010), which governs ion tolerance in a calcineurin‐dependent manner (Cramer *et al*., 2008; Soriani *et al*., 2008), was found to be more tolerant to alkaline pH than an *A*. *nidulans ΔcrzA* mutant (Fig. 1A and C). In stark contrast to an *A*. *nidulans ΔcrzA* mutant, where radial growth at alkaline pH was reduced by ∼ 90% relative to acidic pH, radial growth of an *A*. *fumigatus ΔcrzA* mutant was only reduced by ∼50% (Fig. 1C). However, compared to the respective nonmutated isolates, growth of both *A*. *fumigatus* and *A*. *nidulans ΔcrzA* mutants was severely reduced in the presence of high (200 mM) extracellular calcium concentrations (Fig. 1A).

### Transcriptional uncoupling of *A. fumigatus* alkaline‐ and calcium tolerance

Interdependency of calcineurin‐ and alkaline‐regulated signalling has been described in *S*. *cerevisiae* where alkaline stress prompts a transient rise in cytosolic calcium (Serrano *et al*., 2002; Viladevall *et al*., 2004). Among 48 mutants identified as FK506‐sensitive in a global phenotypic screen, 42% also had an alkaline‐sensitive phenotype, and alkalinity is one of several stressors which prompt relocalisation of *S*. *cerevisiae* Crz1p from the cytoplasm to the nucleus of alkaline‐adapting cells (Ruiz *et al*., 2008). Comparative analysis of alkaline‐ and calcium‐responsive fungal transcriptomes reveals calcineurin dependence of 10% (*n* = 27) of 266 alkaline‐induced genes in transcriptomes in *S*. *cerevisiae* (Viladevall *et al*., 2004) and calcium‐responsive transcription of 11% (*n* = 51) of 453 alkaline‐responsive genes in *C*. *albicans* (Bensen *et al*., 2004; Karababa *et al*., 2006).

To identify commonalities between *A*. *fumigatus* alkaline‐ and calcium‐mediated signalling, we analysed in parallel the temporal transcriptional response of *A*. *fumigatus* following exposure to alkaline shift or elevated extracellular calcium. We characterised the response to alkaline or calcium shift at 5, 15, 30, 45 and 60 min following transfer of freshly cultured germlings (14 h culture) from *Aspergillus* minimal medium (MM) pH 5.0, to MM plus 200 mM calcium chloride (pH 5.0) or MM pH 8.0 (100 mM Tris‐HCl pH 8.0). A common reference hybridization scheme was adopted, whereby temporal alterations (5, 15, 30, 45 and 60 min) in transcript abundance relative to a common time zero (unshifted) sample, were quantified, including technical replications for each time‐point and condition via a dye swapped cohybridisation. Thus, a total of 20 cohybridisation datasets were generated for the analysis.

**Figure 2:**
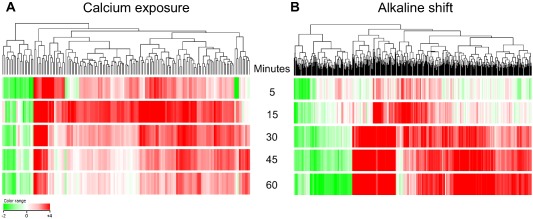
Magnitude and longevity of *A*. *fumigatus* transcriptional responses to calcium exposure and alkaline shift. Temporal profiles of gene expression among (A) calcium‐ and (B) alkaline‐responsive gene cohorts. The transcriptional response to heightened extracellular [Ca^2+^] is swift and transient, showing a peak in the number of differentially expressed genes at 15 min postshift. On the contrary, the transcriptional response to heightened extracellular pH continues throughout the time course of investigation (5–60 min). Red and green indicate up‐ and down‐regulated genes respectively. Lists of differentially regulated genes are provided in Supporting Information Tables S1 and S2.

The dynamics of the transcriptional responses are graphically represented in Fig. 2 which documents the temporal basis of transcriptional reprogramming and details the overall distribution of log_2_ ratios, relative to unshifted sample, in response to each of the alkaline (Fig. 2A) and calcium (Fig. 2B) shifts. The fidelity of microarray data, and biological reproducibility were assessed by comparison to targeted analyses of upregulated, nonregulated and downregulated transcripts by quantitative PCR analysis using independently isolated RNA samples as biological replicates (Supporting Information Fig. S2).

The magnitude and longevity of the resulting transcriptional responses differed greatly. The transcriptional response to calcium exposure is swift, reaching maximal amplitude at 15 min postshift, and stabilises within 30 min of treatment despite continued elevation of extracellular calcium concentrations (Fig. 2 and Supporting Information Table S1). In total the transcript levels of 135 and 24 genes were found to significantly increase or decrease at one or more time points of the analysis respectively (Supporting Information Table S1). Maximal differential was observed at 15 min postshift where 113 genes were upregulated, 37% (*n* = 42) of which were exclusive to the 15 min time‐point. Seven genes remained upregulated throughout the entire time course. Key contributors to maintenance of calcium homeostasis are revealed amongst the upregulated and downregulated gene cohorts including five known or putative calcium transporters amongst the upregulated gene cohort and two amongst the predicted downregulated gene functions.

**Figure 3:**
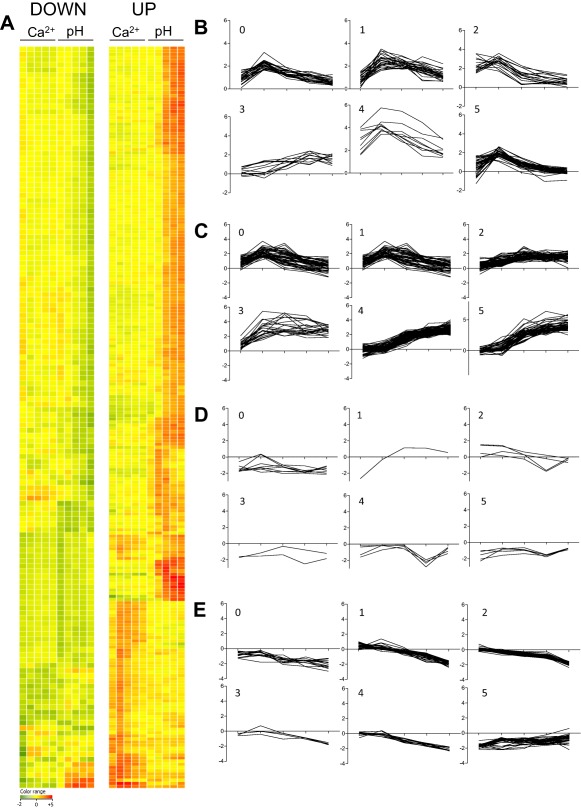
Comparative analysis of *A*. *fumigatus* alkaline‐ and calcium‐responsive transcriptomes. A. Hierarchical clustering of genes responding significantly (log_2_ ratio ≥ ± 1.5) to alkaline shift (pH 8.0) or calcium exposure (200 mM CaCl_2_). K‐means clustering of genes up‐regulated (log_2_ ratio ≥ ± 1.5) in response to (B) 200 mM CaCl_2_ or (C) pH 8.0. K‐means clustering of genes down‐regulated (log_2_ ratio ≥ ± 1.5) in response to (D) 200 mM CaCl_2_ or (E) pH 8.0. Red and green indicate up‐ and down‐regulated genes respectively. Lists of genes, by cluster, are provided in Supporting Information Tables S4–S7.

In response to alkaline extracellular pH, 384 and 126 genes were found to significantly increase or decrease at one or more time points of the analysis respectively (Supporting Information Table S2). In contrast to heightened extracellular calcium concentrations, extracellular alkalinity prompted a gradual and prolonged increase in the number of upregulated genes, which was unabated at the 60 min time‐point (Fig. 2). Comparative analysis of the calcium‐ and alkaline‐responsive transcriptomes (Fig. 3) revealed that differential expression profiles common to both responses involved 32 genes (Supporting Information Table S3). Even within this cohort, however, timescales and directionality of responses differed markedly for all genes (Supporting Information Fig. S3). Our transcriptomic analyses therefore reveal highly discrete adaptation mechanisms to calcium and alkaline exposure in *A*. *fumigatus*.

### Discrete nuclear localisation dynamics for the calcium‐responsive CrzA and pH‐responsive PacC transcription factors in response to alkaline or calcium challenge

**Figure 4:**
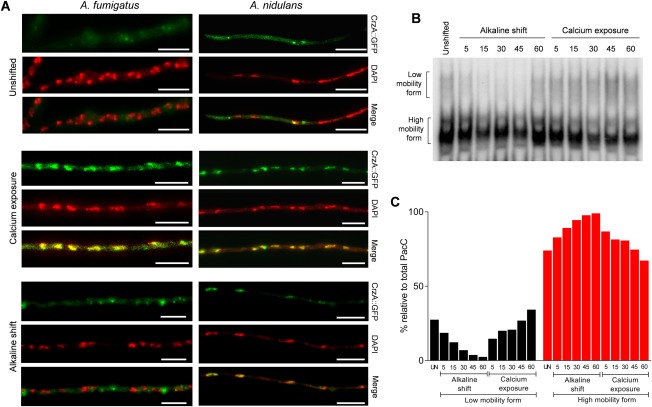
*A*. *fumigatus* and *A*. *nidulans* CrzA nuclear occupancy, in response to heightened extracellular calcium or pH. A. Visualisation by time lapse fluorescence microscopy of *A*. *nidulans* and *A*. *fumigatus* CrzA::GFP recruitment following shift for 5 min to either a high Ca^2+^‐containing medium (200 mM CaCl_2_) or an alkaline medium (pH 8.0). Ca^2+^ exposure induced CrzA::GFP recruitment to nuclei (stained with DAPI); Scale bar = 10 μm. B. Low mobility and high mobility forms of PacC are indicated on the left side. C. Densitometry plot of EMSA data expressed, per complex, as a function of total PacC protein.

The *A*. *fumigatus* CrzA transcription factor is required for calcium tolerance *in vitro* and *ΔcrzA* mutants are significantly attenuated for virulence in a neutropenic murine model of pulmonary aspergillosis (Steinbach *et al*., 2006; Soriani *et al*., 2008). In *S*. *cerevisiae*, *C*. *albicans*, *C*. *neoformans*, *A*. *nidulans* and *A*. *fumigatus* translocation of Crz transcription factors, from the cytoplasm, to the nucleus of stress‐adapting fungal cells occurs in response to heightened extracellular calcium (Stathopoulos‐Gerontides *et al*., 1999; Santos and de Larrinoa, 2005; Karababa *et al*., 2006; Ruiz *et al*., 2008; Soriani *et al*., 2008; Lev *et al*., 2012). In *S*. *cerevisiae, C*. *albicans* and *A*. *nidulans*, alkaline stress also results in a transient elevation of cytoplasmic [Ca^2+^] sufficient to prompt nuclear localisation of Crz1p (Ruiz *et al*., 2008; Hernández‐Ortiz and Espeso, 2013), thereby providing a mechanism for functional coupling of alkaline‐ and calcium‐mediated signalling and gene expression. In order to address the relevance of *A*. *fumigatus* CrzA for alkaline adaptation, we utilised a previously constructed *A*. *fumigatus* CEA10 derivative strain expressing a CrzA::GFP fusion protein (Soriani *et al*., 2008). The nuclear recruitment of *A*. *fumigatus* and *A*. *nidulans* CrzA::GFP was visualised before and after a shift to either a high Ca^2+^‐containing medium (200 mM CaCl_2_) or an alkaline medium (pH 8.0) prior to DAPI staining and fluorescence microscopy. Similarly to what has been previously reported in *A*. *nidulans* (Hernández‐Ortiz and Espeso, 2013) Ca^2+^ exposure induced, in both species, significant CrzA::GFP recruitment to nuclei, with the level of nuclear residence peaking at 5 min post‐treatment and decreasing thereafter (Fig. 4A). However, response to alkaline shift differed between the two species as contrary to our own (Fig. 4A), and previous (Hernández‐Ortiz and Espeso, 2013), observations in *A*. *nidulans* alkaline shift did not induce significant *Af*CrzA::GFP recruitment to nuclei within the timeframe analysed. Under dual calcium and alkaline exposure *A*. *fumigatus* CrzA is translocated to the nucleus of the cell in a manner identical to that we previously observed under calcium exposure (Supporting Information Fig. S3). Nuclear translocation of CrzA is, therefore, neither diminished nor enhanced by alkaline exposure lending further support to the hypothesis that alkaline‐ and calcium‐mediated signalling occur in a mutually independent manner. Given the absence of a CrzA transcriptional signature (Soriani *et al*., 2010) in response to alkaline stress (Fig. 3 and Supporting Information Data S1), we conclude either that nuclear occupancy alone is insufficient for functionality of *A*. *fumigatus* CrzA, or that a threshold quantity of nuclear CrzA is required to initiate a transcriptional response.

The *A*. *fumigatus* PacC transcription factor undergoes pH dependent proteolytic cleavage in order to enter the nucleus and orchestrate alkaline adaptation (Peñalva *et al*., 2008; Bertuzzi *et al*., 2014). Accordingly, by electrophoretic mobility shift assay (EMSA) several retardation complexes can be detected and their relative quantities vary upon acidic and alkaline growth conditions. The abundance of the PacC retardation complexes was visualised and quantified before and after a shift to either a high Ca^2+^‐containing medium (200 mM CaCl_2_) or an alkaline medium (pH 8.0) for 5, 15, 30, 45 and 60 min (Fig. 4B and C). Relative to the unshifted control, the processed, activated form of PacC (high mobility) increased in abundance upon shifting to alkaline conditions, whist no increase was detected upon shift to a high Ca^2+^‐containing medium. Taken together, the discrepant extent of CrzA nuclear occupancy and PacC proteolytic processing (Fig. 4) and the discordant transcriptional responses (Supporting Information Data S1 and S2) indicate significantly different cellular bases for alkaline‐ and calcium‐mediated signalling in *A*. *fumigatus*.

### Transcriptional regulation of an exemplar alkaline‐responsive gene, Af*ena1*, occurs independently of calcineurin signalling in *A. fumigatus*

**Figure 5:**
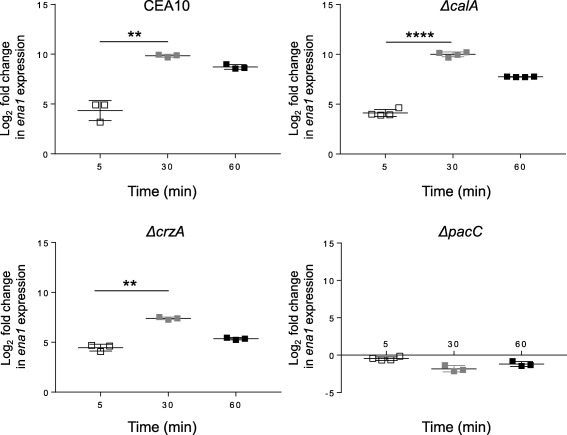
Genetic perturbation of *ena1* transcriptional regulation. The expression of *ena1* was quantified by qPCR using RNA extracted from alkaline‐exposed germlings of four different *A*. *fumigatus* strains at three different time points (5, 30 and 60 min): CEA10, *ΔcalA*, *ΔcrzA* and *ΔpacC*. The Y‐axis represents the log_2_ fold change, normalised to ß‐tubulin, determined by comparison to the untreated sample. *n* = 3–4, error bars = SEM.

Among genes responding immediately to alkaline pH, our microarray analyses revealed rapid up‐regulation of a putative *ENA1* homologue AFUA_6G03690 (Supporting Information Tables S2 and S5). In *S*. *cerevisiae* the family of ENA‐type sodium ATPases govern detoxification of the cytosol by active extrusion of cations. Under alkaline conditions, since the proton gradient used by fungal cells to exchange intracellular cations for extracellular protons is reversed, Ena1p functionality becomes essential for cellular survival, and as such, ENA‐type sodium ATPases are prototypically alkaline‐responsive genes (Haro *et al*., 1991). In *S*. *cerevisiae*, *A*. *nidulans* and *C*. *albicans*, *ENA1* homologues are subject to convergent, and/or parallel regulation by multiple transcription factors acting co‐operatively to protect the cell from the ionic challenges posed by high osmolarity, cationic stress and pH fluctuations (Platara *et al*., 2006; Kullas *et al*., 2007; Spielvogel *et al*., 2008; Petrezsélyová *et al*., 2016). To assess the relative contributions of pH‐ and calcineurin‐mediated signalling to Af*ena1* regulation, we performed a targeted analysis of Af*ena1* transcription using quantitative PCR and RNA extracted from alkaline‐exposed *A*. *fumigatus* germlings (Fig. 5). In agreement with our phenotypic (Fig. 1), transcriptional (Fig. 3) and cellular (Fig. 4) analyses we found that Af*ena1* transcription, in response to high pH, occurs independently of calcineurin and CrzA (Fig. 5). Thus, major drivers of alkaline adaptation are regulated independently of calcineurin signalling in this pathogenic mould. To further interrogate the molecular basis of alkaline‐responsive *ena1* regulation we assessed Af*ena1* expression in a *ΔpacC* mutant (Amich *et al*., 2009) lacking the pH‐responsive transcription factor PacC. In this isolate alkaline‐responsive upregulation of Af*ena1* transcription is completely abrogated (Fig. 5). The observed unilateral dependency of Af*ena1* regulation upon PacC is highly supportive of the theory that in *A*. *fumigatus*, alkaline‐responsive gene regulation and alkaline tolerant growth circumvent calcineurin signalling.

## Discussion

The ability to tolerate and grow in alkaline conditions is essential for virulence of multiple fungal pathogens of man (Peñalva *et al*., 2008), and an important prospect for therapeutic inhibition of infectious growth (Abadio *et al*., 2011; Bignell, 2012; Cornet and Gaillardin, 2014). In *S*. *cerevisiae*, *A*. *nidulans*, *C*. *albicans* and *C*. *neoformans* (Odom *et al*., 1997; Bader *et al*., 2003; Viladevall *et al*., 2004; Spielvogel *et al*., 2008; Hernández‐Ortiz and Espeso, 2013) adaptation to alkaline stress requires a calcium‐mediated signal, transmitted via the highly conserved calcium‐dependent phosphatase, calcineurin. Agents which target highly conserved nodes of fungal alkaline adaptation might, therefore, potentiate the inhibitory activity of calcineurin inhibitors, the antifungal potency of which cannot be harnessed in human patients due to their non‐selective mode of toxicity. In this study, we elucidated, using chemical and genetic perturbations, the regulatory hierarchy which governs highly conserved regulators of alkaline adaptation and calcium tolerance in *A*. *fumigatus*. Our analysis revealed that the transcriptional circuitry has evolved in *A*. *fumigatus* to robustly exploit alkaline‐responsive genes, both *in vitro* and *in* vivo, independently of calcium‐dependent hyphal growth and morphogenesis.

Using a membrane‐impermeable chelator of calcium ions to prevent acquisition of extracellular calcium, we discovered an absolute requirement for calcium for *A*. *fumigatus* growth (Fig. 1A). This finding, although novel for *A*. *fumigatus*, is in keeping with those applicable to other fungi growing by hyphal extension. The polarised extension of tip‐growing fungal cells requires the precise spatial and temporal maintenance of cell wall deposition, coupled with an expansive force derived from turgor pressure or the cytoskeleton (Jackson and Heath, 1993), a process dependent upon highly localised calcium gradients (Silverman‐Gavrila and Lew, 2003). In the model ascomycete, *Neurospora crassa*, hyphal morphology becomes highly aberrant at low Ca^2+^ concentrations whereby branching frequency, hyphal width and apical dominance are dysregulated (Jackson and Heath, 1993). The same effect is observed in *A*. *fumigatus* in mutants lacking calcineurin subunits (Steinbach *et al*., 2006; da Silva Ferreira *et al*., 2007). Subapical removal of calcium via efflux pumps, and/or organellar sequestration, is presumed to maintain the precise orchestration of calcium signalling required for tip‐directed hyphal growth (Jackson and Heath, 1993), this could be disrupted by scarcity of extracellular calcium if the impetus for inward diffusion, or active uptake, of calcium were lost. If so, we could conclude from our data that, under calcium‐limiting conditions, *A*. *fumigatus* is unable to liberate sufficient calcium from internal stores to support hyphal growth and/or is unable to appropriately establish localised calcium gradients when available calcium is restricted to that derivable from internal supplies. Our findings therefore suggest that growth of *A*. *fumigatus* hyphae requires localised influx of extracellular Ca^2+^ in the tip region, and that *A*. *fumigatus* is incapable of supporting hyphal growth when calcium supply is severely limited. Extracellular Ca^2+^ is also required for hyphal extension to occur in *N*. *crassa and Fusarium graminearum* (Schmid and Harold, 1988; Robson *et al*., 1991), however, previous studies, using self‐referencing ion‐selective probes, have failed to demonstrate net Ca^2+^ influx during *N*. *crassa* hyphal growth (Lew, 1999) and direct manipulation of the membrane potential does not affect hyphal growth rate (Silverman‐Gavrila and Lew, 2003). In agreement with the latter finding, we found *A*. *fumigatus* growth under limiting calcium to be insensitive to extremes of extracellular pH (Fig. 1A), a result which would not be expected if calcium uptake were significantly influenced by proton motive force. The mechanistic basis of calcium uptake during *A*. *fumigatus* growth remains unknown but the essential requirement for calcium during filamentous growth of this pathogen would highlight a credible opportunity for selective therapeutic intervention if a fungus‐specific calcium uptake mechanism is found to exist. Given that *A*. *fumigatus* hyphae elicit severe tissue damage in mammalian lung tissue a plausible strategy for preventing infectious growth would be to block the inward migration of calcium ions.

To study the transcriptional consequences of transiently elevated cytosolic [Ca^2+^] we exposed *A*. *fumigatus* to high concentrations of extracellular calcium. We employed a concentration of extracellular calcium (200 mM) proven to evoke (a) expression of stretch‐activated calcium channels in *A*. *fumigatus* (de Castro *et al*., 2014) and (b) to elicit nuclear transmigration of the calcium‐responsive family of Crz transcription factors, both in *A*. *fumigatus* (Soriani *et al*., 2008) and in analogous studies of other fungi (Lev *et al*., 2012). The biological relevance of this stimulus in our study was substantiated by resultant CrzA::GFP nuclear translocation (Fig. 4) and by the highly evident overlap between our data and that delivered by a previous study of the CrzA‐dependent regulon such that 14 out of 32 genes previously found to be downregulated in a CrzA null mutant (Soriani *et al*., 2010) were also identified as calcium upregulated in our study (Supporting Information Table S1). Included in this cohort are two of the three *A*. *fumigatus* gene products annotated as calcium‐translocating P‐ATPases, PmcB (AFUA_3G10690) and PmcA (AFUA_1G10880), the latter of which has recently been identified as essential for pathogenicity. Our study delivers the first characterisation of an alkaline‐adapting transcriptome for *A*. *fumigatus*, revealing that alkaline‐responsive transcription exhibits markedly different kinetics to that observed during calcium‐responsive growth (Fig. 3).

Concordant with the theory that moderation of proton homeostasis plays a critical role in pH adaptation the alkaline transcriptome is defined by physiological reprogramming of cellular metabolism and transport activities whereby the majority of differentially expressed genes participate in some aspect of cellular metabolism, or transport. Negligible concordance between alkaline‐ and calcium‐responsive gene expression was detectable, thereby diminishing the relevance of our initial hypothesis that co‐operative alkaline and calcium signalling is required during *A*. *fumigatus* infection. The relevance of alkaline‐ and calcium‐rich microenvironments during lung infection is demonstrable from comparative analysis of our previous transcriptomic studies of murine aspergillosis (Bertuzzi *et al*., 2014), and the datasets generated in this study wherein 53% (*n* = 271) and 45% (*n* = 72), respectively, of the alkaline‐ and calcium‐responsive gene cohorts are captured. The functional relevance of both signalling pathways during infection of the leukopenic mouse is furthermore underscored by strict requirements for both CrzA and PacC for full virulence of *A*. *fumigatus* (Steinbach *et al*., 2006; Soriani *et al*., 2008; Bertuzzi *et al*., 2014). On the basis of our analyses of the regulatory hierarchy in this pathogen it is highly likely that alkaline‐ and calcium‐adaptation are occurring simultaneously during infection, and are regulated in parallel, by PacC and CrzA respectively. Concordant with a differential functional significance of the two signalling mechanisms during infection, PacC has been characterised as a critical regulator of tissue invasion in the murine host (Bertuzzi *et al*., 2014), whilst CrzA null mutants exhibit an entirely different infection phenotype, most likely resulting from a failure to grow within the host due to cation toxicity.

In *A*. *fumigatus*, the transcriptional response to alkalinisation involves immediate upregulation of the gene encoding the Ena1 sodium H‐ATPase indicating cation extrusion as the initial cellular response to high pH (Supporting Information Table S2). This observation correlates with those reported for *S*. *cerevisiae*, *C*. *albicans* and *C*. *neoformans* (Odom *et al*., 1997; Serrano *et al*., 2002; Bader *et al*., 2003; Viladevall *et al*., 2004; Kullas *et al*., 2007; Ruiz *et al*., 2008; Wang *et al*., 2011) and suggests that in *A*. *fumigatus* also, intracellular cations are toxic to the fungal cell at high pH. Concordant with this prediction, and with the finding that Ena1 is explicitly governed by PacC during alkaline adaptation (Fig. 5), *A*. *fumigatus* PacC null mutants exhibit cation‐sensitive phenotypes at alkaline pH (Bertuzzi *et al*., in preparation). However, in contrast to *A*. *nidulans* where combined sodium chloride and alkaline stresses are required for *enaA^AN6642^* gene induction (Spielvogel *et al*., 2008) upregulation of Af*ena1* is observed under pH stress at mM cation concentrations. In further support of a differential mechanistic basis for *ena1* regulation in *A*. *fumigatus* and *A*. *nidulans*, our data reveal that unlike *A*. *nidulans Δcrz* mutants, those lacking the homologous *A*. *fumigatus* transcription factor CrzA are not alkaline sensitive (Fig. 1C and D). Of further note is alkaline‐, but not calcium‐dependency of the *A*. *fumigatus* pho89p homologue (AFUA_3G03010) which is a demonstrated target of Crz1p in yeast and is expressed in a coregulated fashion with the Ena1 Na^+^‐ATPase to allows their functional coupling under high‐pH stress (Serra‐Cardona *et al*., 2014). In *S*. *cerevisiae*, the *PHO89* promoter is regulated by Crz1p and negatively regulated by several Snf1p and Rim101p‐dependent repressors. This regulatory paradigm mimics that of Sc*ENA1* expression and it is proposed in yeast that the activities of Pho89p and Ena1p are functionally coordinated to sustain growth in an alkaline environment. Our data suggest a similar functional scenario in *A*. *fumigatus* where co‐expression of *pho*89 and *ena1* expression has been maintained to facilitate alkaline tolerance, but in a regulatory environment which circumvents CrzA‐mediated signalling.

Similar to *A*. *nidulans*, the *A*. *fumigatus* gene encoding PacC (AFUA_3G11970), appears to be alkaline‐expressed but the gene encoding the arrestin‐like activator of PacC‐mediated signalling RIM8/PalF (AFUA_4G09650), which in *A*. *nidulans* is markedly downregulated by alkaline pH (Bussink *et al*., 2015), appears not to be transcribed in a pH‐responsive manner. It has been postulated for *A*. *nidulans* that repression of acid‐expressed *palF*, specifying the Pal pathway arrestin, probably by PacC^27^ and/or PacC^53^, prevents an escalating alkaline pH response. The absence of such a signature in *A*. *fumigatus* might contribute to the significantly heightened quantities of processed *A*. *fumigatus* PacC which we routinely observe in mobility shift assays (this study and Bertuzzi *et al*., 2014).

In *A*. *nidulans* a further transcription factor, SltA, is involved in cation homoeostasis and detoxification. *ΔsltA* mutants have alkaline‐ and cation‐sensitive phenotypes and fail to appropriately regulate expression of *ena* homologues. It is, therefore, possible that the *A*. *fumigatus* SltA homologue also plays a role in alkaline tolerance. In the present study we intentionally focused upon highly evolutionarily conserved transcription factors because were interested in therapeutically relevant diversification of fungal regulatory circuits. In terms of informing broad spectrum antifungal strategy this approach has more relevance than focusing on species‐specific regulatory traits. SltA is not a highly conserved protein, indeed it is restricted to the Ascomycota phylum, and is absent in yeasts, with more remote orthologues (having less than 49% identity) occurring in *Histoplasma capsulatum*, and various Cryptococcus and Penicillium species (as stated in Spielvogel *et al*., 2008). Further, our ability to perform directly comparative analyses of *ENA* gene expression in *A*. *fumigatus* and *A*. *nidulans* would be hampered by the necessity in *A*. *nidulans* to impose a combination of elevated concentrations of Na^+^ and alkaline pH (Spielvogel *et al*. used 1M NaCl in their study). Amongst a 401‐member library of *A*. *fumigatus* transcription factor null mutants under study in our laboratory a minimum of 10 other transcription factors demonstrate alkaline‐sensitive phenotypes (unpublished). A robust analysis of the impact of their integrated activity upon alkaline tolerance will undoubtedly arise from future network analyses.

In *A*. *fumigatus* nuclear recruitment of CrzA is observed immediately after calcium exposure; however alkaline adaptation does not involve nuclear accumulation of CrzA (Fig. 4). In *S*. *cerevisiae*, the rapid alkaline‐mediated response of the *ENA1* promoter is congruent with a substantial presence of the calcineurin‐activated transcription factor Crz1p in the nucleus after 2.5–5 min of exposure to alkaline stress (Serrano *et al*., 2002; Ruiz *et al*., 2008) and in *A*. *nidulans* alkalinisation of the media induces nuclear accumulation of CrzA after 1–10 min of exposure (Hernández‐Ortiz and Espeso, 2017). In contrast, we did not observe nuclear occupancy by CrzA::GFP in *A*. *fumigatus* after alkaline exposure for 5 or 60 min (Fig. 4A). Moreover, although the timing of *A*. *fumigatus* CrzA nuclear recruitment correlates positively with that of Af*ena1* alkaline‐induced transcriptional upregulation, Af*ena1* expression was not found to alter in response to calcium exposure (Supporting Information Tables S1 and S2 and Fig. S2A). Furthermore, comparisons of the previously characterised *A*. *fumigatus* CrzA‐dependent transcriptional regulon (Soriani *et al*., 2008) to those of our *in vitro* analyses of alkaline‐ and calcium‐responsive gene expression (Supporting Information Table S1 and S2) identifies calcium‐mediated upregulation of *n* = 22 CrzA‐dependent genes but does not identify a CrzA‐dependent regulon amongst alkaline‐responsive *A*. *fumigatus* genes. Taken together, the absence of a CrzA transcriptional signature amongst alkaline‐responsive genes (this study and Soriani *et al*., 2008) and the absolute dependency of Af*ena1* transcription upon PacC (Supporting Information Tables S1 and S2 and Fig. S2A), provide a compelling case for PacC being the sole regulator of alkaline‐responsive Af*ena1* transcription in *A*. *fumigatus*. However, this regulatory pattern is not necessarily extensive to other *A*. *fumigatus* genes.

**Figure 6:**
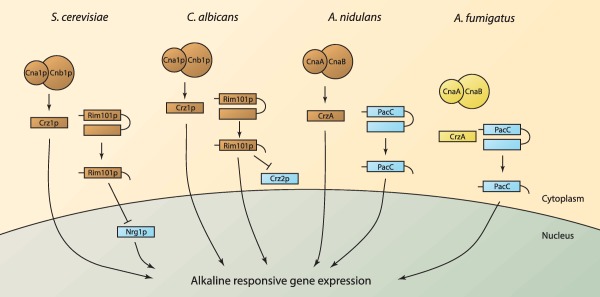
Distinct mechanistic interdependency of pH‐ and calcium‐mediated signalling in fungal pathogens of man. Relative to *A*. *nidulans, C*. *albicans* and *S*. *cerevisiae*, significant regulatory divergence exists in in the pathogenic mould *A*. *fumigatus* as demonstrated by genomic, transcriptomic, phenotypic, cellular and chemical genetic analyses. Brown: Required for both alkaline and calcium tolerance. Blue: required for alkaline tolerance. Yellow: required for calcium tolerance.

Via a series of phenotypic, genetic, chemical genetic, cellular and transcriptomic analyses our study demonstrates that, relative to yeasts, and the rarely pathogenic ascomycete *A*. *nidulans*, pH‐ and calcium‐mediated signalling are functionally uncoupled in *A*. *fumigatus* such that alkaline‐responsive gene expression occurs independently of calcineurin. In contrast to orchestration of alkaline‐responsive gene expression in *S*. *cerevisiae*, *A*. *nidulans* and *C*. *albicans* (Serrano *et al*., 2002; Bensen *et al*., 2004; Viladevall *et al*., 2004; Wang *et al*., 2011; Hernández‐Ortiz and Espeso, 2013) nuclear translocation of the *A*. *fumigatus* CrzA transcription factor is not involved in the response to alkaline exposure and is not required for alkaline tolerance (Fig. 6). In terms of pathogenicity this profound regulatory distinction might promote mammalian infections by (a) alleviating cellular demands upon calcium‐mediated signalling, which itself plays a critical role in mediating invasive *A*. *fumigatus* hyphal growth, and (b) by broadening, compared to the lesser pathogenic ascomycete, *A*. *nidulans*, the repertoire of tolerable stresses, an important trait for pathogenicity in the mammalian host.

## Experimental procedures

### Strains and media

*A*. *fumigatus* strains used in this study are the clinical isolates CEA10 (Monod *et al*., 1993), Af237 (Tang *et al*., 1993), and derivatives thereof, and laboratory isolates H515 (Brown *et al*., 2000), *ΔcalA* (da Silva Ferreira *et al*., 2007), *ΔcrzA* (Soriani *et al*., 2008), CrzA::GFP (Soriani *et al*., 2008) and *ΔpacC* (Amich *et al*., 2009). *A*. *nidulans* strains used in this study are HHF27A wild type prototroph (Findon *et al*., 2010), HHF27L *ΔcrzA* (*crzAΔ::pyr‐4^Nc^*, kind gift of Herb Arnst) and MAD3021 (*pyrG89, pabaB22, argB2, nkuAΔ::argB, crzA::gfp/pyrGAf*) (Hernández‐Ortiz and Espeso, 2013). Unless otherwise stated, media used were Aspergillus Minimal Media (MM) or Aspergillus Complete Media (ACM) (Pontecorvo *et al*., 1953). When indicated, p amino benzoic acid (PABA) was added at a final concentration of 4 mg L^−1^. A standard growth temperature of 37°C was employed throughout. Calcineurin inhibitor FK506 and the calcium chelator 1,2‐bis(o‐aminophenoxy)ethane‐*N*,*N*,*N*',*N*'‐tetraacetic acid (BAPTA) were purchased from Sigma UK.

### Aspergillus phenotypic analysis

*A*. *fumigatus* spores were harvested from cultures grown on solid ACM and counted using a hemocytometer. Serial dilutions of 10^2^, 10^3^, 10^4^ conidia were inoculated onto supplemented MM and incubated at 37°C for 48 h. Images were captured using a Nikon digital camera and the 10^3^ conidia dilution was chosen to represent the results. Images were processed using the free software Irfanview. Radial growth was measured by assessing colony diameter in cm.

### RNA isolation

RNA was isolated from *A*. *fumigatus* mycelia grown in supplemented liquid MM (pH 5.0 with 100 mM glycolic acid pH 5.0) for 16 h. After 16 h, calcium signalling was induced by a shift to MM (pH 5.0) plus 200 mM CaCl_2_ and alkaline signalling was induced by a shift to MM plus 100 mM Tris‐HCl pH 8.0. Mycelia were exposed to the shift conditions for 5, 15, 30, 45 and 60 min, harvested, snap‐frozen in liquid nitrogen and disrupted by grinding. A common time zero (unshifted) sample was also collected for RNA extraction. Total RNA was extracted with RNA‐Bee (Amsbio, UK) following the manufacturer protocol. About 1 μg of total RNA was treated with RNase‐free DNaseI (Invitrogen, UK) and RNA quality was determined using a Nanodrop ND‐1000 spectrophotometer and agarose gel electrophoresis.

### DNA microarray methods

We used the *A*. *fumigatus* oligonucleotide slides version 3 for microarray hybridizations (for details see http://pfgrc.tigr.org/slide_html/microarray_descriptions.shtml) using a common reference hybridization scheme whereby temporal alterations (5, 15 30, 45 and 60 min) in transcript abundance relative to a common time zero (unshifted) sample, were quantified, including technical replications for each time‐point and condition via a dye swapped cohybridisation. Thus, a total of 20 cohybridisation datasets were generated for the analysis. The RNA samples were shipped to the microarray facility at the J. Craig Venter Institute (Rockville, MD) for further analysis using the standardized facility protocols available at http://www.tm4.org/spotfinder.html. Briefly, 30 μg of total RNA was mixed with 20 μg of Random Primer Hexamers (GE Healthcare), 100 pmol of oligo dT (Invitrogen) and 40 U of RNAseOUT (Invitrogen) in a volume of 13 μl and heated to 70°C for 5 min. The tubes were briefly chilled to 4°C for 5 min and mixed with 5 μl of 5X first‐strand buffer (Invitrogen), 2 μl of DTT (100 mM, Invitrogen), 2 μl of 25 mM dNTP, 2 μl of SuperScript RT (Clontech) and 1 μl of Cy3‐dUTP (25 nmol) or Cy5‐dUTP (25 nmol). After 16 h of incubation at 42°C, RNA was hydrolysed by adding 2.5 μl EDTA (0.5 M, pH 8) and 5 μl NaOH (1 M) following incubation at 37°C for 40 min. The resulting first strand cDNA was purified and concentrated using a MiniElute column (Qiagen) according to the manufacturer instructions.

Probes were heated at 95°C for 5 min prior to addition to the array. Slides were hybridized for 12–16 h at 42°C in a Gene‐Tac Hybridization Station (Genomic Solutions) and washed in 2X SSC 0.1% (v/v) SDS, 0.1X SSC 0.1% (v/v) SDS and 0.1X SSC. Slides were then subjected to fluorescent detection with a GMS 418 Array Scanner (Affymetrix Inc., Santa Clara, CA) and the TIFF images generated were analysed using TIGR Spotfinder (http://www.jcvi.org/cms/research/software/) to obtain relative transcript levels. Data were normalized using a local regression technique LOWESS (LOcally WEighted Scatterplot Smoothing) for hybridizations with RNA‐based samples and SD regularization of the Cy5/Cy3 ratio across all sectors (blocks) of the array using a software tool MIDAS (http://www.jcvi.org/cms/research/software/). The resulting data were averaged from duplicate genes on each array and from dye‐swap hybridizations for each experiment. Differentially expressed genes at the 95% confidence level were determined using intensity‐dependent *Z*‐scores (with *Z* = 1.96) as implemented in MIDAS and the union of all genes identified at each time point were considered significant in this experiment. The resulting data were organized and visualized based on similar expression vectors in genes using Euclidean distance and hierarchical clustering with average linkage clustering method to view the whole data set using the Genespring software (Agilent). Raw data have been deposited in the Gene Expression Omnibus (GEO) (http://www.ncbi.nlm.nih.gov/geo/) under accession number GSE60786.

### Microscopy analysis

Five hundred conidia ml^−1^ of *A*. *fumigatus* expressing CrzA::GFP (Soriani *et al*., 2008) were grown in 8‐well slide culture chambers for 16 h in filtered minimal medium (pH 5.0 with 100 mM glycolic acid pH 5.0) at 37°C. After 16 h, calcium signalling was induced by a shift to MM (pH 5.0 with 100 mM glycolic acid pH 5.0) plus 200 mM CaCl_2_ and alkaline signalling was induced by a shift to MM plus 100 mM Tris‐HCl pH 8.0. Calcium exposure and alkaline shift were performed for 5 and 60 min. Fixing of the samples was performed using fixative solution (3.7% v/v formaldehyde, 50 mM w/v sodium phosphate buffer pH 7.0, 0.2% v/v Triton X‐100) for 30 min at room temperature. After rinsing with PBS, samples were incubated for 5 min in a solution with 100 ng ml–1 of DAPI (4′,6‐diamino‐2‐phenyylindole, Sigma) for nuclear staining. Samples were then washed with PBS buffer for 5–10 min at room temperature and visualized using a Nikon Eclipse TE2000E microscope with DIC optics, a 606 (1.3 NA) plan fluor objective and equipped with an ORCA‐ER CCD camera (Hamamatsu, Welwyn Garden City, UK) driven by the MetaMorph NX1.1 software for image acquisition. For DAPI, a Nikon UV‐2A filter cube (excitation filter 355/15 nm BP, dichroic mirror 400 nm LP, emission filter 420 nm LP) was used. For GFP, a Nikon B‐2A filter cube (excitation filter 470/20 nm BP, dichroic mirror 500 nm LP, emission filter 515 nm LP) was used. Images were processed and analyzed using the software ImageJ (http://rsbweb.nih.gov/ij/). Images show maximum intensity projections with inverted look‐up tables (LUTs).

### Quantitative real time PCR (RT‐qPCR)

RNA was generated using the previously described protocol. Four μg of total RNA were treated with DNaseI (Invitrogen) in order to remove genomic DNA contamination. Subsequently, 1 μg of DNaseI treated total RNA was reverse transcribed into cDNA using SuperScriptIII Reverse Transcriptase (Invitrogen) as per manufacturer's instructions. RT‐qPCR validation of the microarray was performed analysing temporal alterations (5, 15 30, 45 and 60 min) in transcript abundance relative to a common time zero (unshifted) sample with technical replications for each time‐point and shift condition. SYBR Green JumpStart Taq Ready Mix (Sigma, UK) was used to perform RT‐qPCR analyses. Probes of an approximate length of 150 nucleotides were designed using the primers listed in Supporting Information Table S8. In all experiments, appropriate negative controls containing no template DNA or RNA were subjected to the same procedure to exclude or detect any possible contamination. All samples were prepared in triplicate and the RT‐qPCR was performed using a Rotor‐Gene RG‐3000 (Corbett Research, UK) with the following thermal cycling parameters: 95°C for 5 s, 58°C for 5 s, 72°C for 8 s, 78°C for 5 s, 81°C for 5 s and 50 times. Data were then analyzed using the RotorGene 6 software and the relative expression values were determined using a previously generated standard curve. The average of the three replicates and the standard deviation were calculated and all values were normalized to the β‐tubulin gene (AFUA_7G00250) values. Fold changes in expression were calculated between the treated and the untreated sample and the log_2_‐transformed values were used for graphical representation.

### Electromobility shift assays

Spores were inoculated at a density of 1x10^6^ to 2x10^6^ spores ml^−1^ in 100 ml of liquid MM (pH 5.0 with 100 mM glycolic acid pH 5.0) and grown for 16 h at 37°C. Next, calcium signalling was induced by a shift to MM (pH 5.0 with 100 mM glycolic acid pH 5.0) plus 200 mM CaCl_2_ and alkaline signalling was induced by a shift to MM plus 100 mM Tris‐HCl pH 8.0. Mycelia were exposed to the shift conditions for 5, 15, 30, 45 and 60 min, harvested, snap‐frozen in liquid nitrogen and lyophilised. Ten mg of protein was extracted from washed mycelia as described previously (Orejas *et al*., 1995). Protein concentrations were determined using the Bradford assay (Bradford, 1976). The *ipnA2* probe was synthesised and labelled as described previously (Orejas *et al*., 1995; Tilburn *et al*., 1995). Densitometry data were obtained using the software ImageJ (http://rsbweb.nih.gov/ij/).

### Statistical analysis of data

GraphPad Prism was used to interpret data and *p* values were calculated using paired t tests. Error bars show the Standard Error of the Mean (SEM) and Standard Deviation (SD) as indicated. * *p* ≤ 0.05, ** *p* ≤ 0.01, *** *p* ≤ 0.001, **** *p* ≤ 0.0001.

## Author contributions

EMB devised the study. EMB and MB wrote the paper. OL, YY, BLM, NF, EAE, DAJ, NDR, WCN revised the manuscript critically. OL, MB, BLM, EAE and EMB designed, performed and analysed the experiments. MB and NDR designed, performed and analysed the microscopy experiments. YY, NF and WCN designed, performed and analysed the microarray experiments. All authors approved the final version of the manuscript.

## Supporting information

Supporting Legends

Supporting Figure S1

Supporting Figure S2

Supporting Figure S3

Supporting Figure S4

Supporting Table 1

Supporting Table 2

Supporting Table 3

Supporting Table 4

Supporting Table 5

Supporting Table 6

Supporting Table 7

Supporting Table 8
