# Supplementary material for: Mutual independence of alkaline‐ and calcium‐mediated signalling in Aspergillus fumigatus refutes the existence of a conserved druggable signalling nexus
Source: Mol Microbiol. 2017 Nov 14;106(6):861–75. doi: 10.1111/mmi.13840 (PMC5725717; doi:10.1111/mmi.13840)
Supplement: Supplementary file 1 — Supporting Legends [file MMI-106-861-s001.docx]

**Title page**

Mutual independence of alkaline- and calcium-mediated signalling in *Aspergillus fumigatus* refutes the existence of a conserved druggable signalling nexus

Omar Loss*^, A^, Margherita Bertuzzi*^, 1^, Yan Yu^2^, Natalie Fedorova^2^, Bethany L McCann^1^, Darius Armstrong-James^3^, Eduardo A Espeso^4^, Nick D Read^1^, William C Nierman^2^, Elaine M Bignell^1^

From 1. Manchester Fungal Infection Group, Division of Infection, Immunity and Respiratory Medicine, University of Manchester, Manchester M13 9NT, United Kingdom, 2. The J. Craig Venter Institute, Rockville, Maryland, USA, 3. Fungal Pathogens Laboratory, National Heart and Lung Institute, Imperial College London SW7 2AY, 4. Department of Molecular and Cellular Biology, Centro de Investigaciones Biologicas (C.S.I.C.), Madrid, Spain

**Running title:** pH and Ca^2+^ signalling in *Aspergillus fumigatus*

*These authors contributed equally to this work

Address correspondence to: Dr. Elaine M Bignell, Manchester Fungal Infection Group, Division of Infection, Immunity and Respiratory Medicine, University of Manchester, Manchester M13 9NT, United Kingdom, Tel: 0044(0)161 275 0678, Email: [elaine.bignell@manchester.ac.uk](mailto:elaine.bignell@manchester.ac.uk)

**Keywords:** Calcium signalling, pH regulation, *Aspergillus fumigatus*, transcription factor

Present address:

A Ipsen Bioinnovation Ltd, Abingdon, UK

**Supplementary material legends**

FIGURE S1. Requirement for extracellular calcium during *A. fumigatus* growth.

Elevation of exogenous [Ca^2+^] by supplementation with 200 mM CaCl_2_ in the presence of 5 mM BAPTA restores hyphal growth.

FIGURE S2. Validation of microarray data using RT-qPCR.

(A) 4 genes, *dprB* (AFUA_6G12180), *anx3.2* (AFUA_2G13890), *ena1* (AFUA_6G03690) and *mid1* (AFUA_5G05840) were selected for the verification of the microarray data. Transcript abundance was quantified via RT-qPCR at time points corresponding to those used for the microarray analysis (5, 15, 30, 45 and 60 minutes after shift to either a high Ca^2+^-containing medium, 200 mM CaCl_2_ or an alkaline medium, pH 8.0). On the Y-axis is represented the log_2_ fold change in expression in the microarray (red) and RT-qPCR (black) analyses, relative to the untreated sample. Values represent the mean of three independent measures. (B) Correlation analysis of the log_2_ fold change in expression in the microarray and RT-qPCR was performed using nonlinear regression, < 0.0001.

FIGURE S3. Hierarchical clustering of genes responding, in this study, significantly (log_2_ ratio ≥ +/- 1.5) to alkaline shift (pH 8.0), or calcium exposure (200 mM CaCl_2_) and which are also differentially regulated during infection in a leukopenic murine model of aspergillosis ([Bertuzzi et al., 2014](file:///J:\Manuscripts%20in%20preparation\Mol%20Mic%202017\REBUTTAL%20Submission\CORRECTED%20VERSION_02082017.docx#_ENREF_6)). Red and green indicate up- and down-regulated genes respectively. List of genes differentially regulated in response to 200 mM CaCl_2_ exposure and in response to a shift to alkaline pH (pH 8.0) is provided in Supplementary Data 3.

FIGURE S4. *A. fumigatus* and *A. nidulans* CrzA nuclear occupancy, in response to heightened extracellular calcium or pH.

(A) Visualisation by time lapse fluorescence microscopy of CrzA::GFP recruitment following shift for 5 or 60 minutes to either a high Ca^2+^-containing medium (200 mM CaCl_2_) or an alkaline medium (pH 8.0). Ca^2+^ exposure induced CrzA::GFP recruitment to nuclei (stained with DAPI), with the level of nuclear occupancy peaking at 5 minutes post-treatment; Scale bar = 10 μm (B) PacC processing after shift to either a high Ca^2+^-containing medium (200 mM CaCl_2_) or an alkaline medium (pH 8.0) by EMSA. Low mobility and high mobility forms of PacC are indicated on the right side. (C) Densitometry plot of EMSA data expressed, per complex, as a function of total PacC protein.

TABLE S1. List of genes differentially regulated in response to 200 mM CaCl_2_ exposure.

Up- and down-regulated genes (log_2_ ratio ≥ +/- 1.5) in response to 200 mM CaCl_2_ exposure for 5, 15, 30, 45 and 60 minutes are annotated. For each time-point in the analysis, the log_2_ fold change in expression relative to the untreated sample is tabulated. The first time-point of significant differential expression and the subsequent time-points at which each gene is differentially expressed is also tabulated.

TABLE S2. List of genes differentially regulated in response to a shift to alkaline pH (pH 8.0).

Up- and down-regulated genes (log_2_ ratio ≥ +/- 1.5) in response to shift to alkaline pH (pH 8.0) for 5, 15, 30, 45 and 60 minutes are annotated. For each time-point in the analysis, the fold change in expression relative to the untreated sample is tabulated. The first time-point of significant differential expression and the subsequent time-points at which each gene is differentially expressed is also tabulated.

TABLE S3. List of genes differentially regulated in response to 200 mM CaCl_2_ exposure and in response to a shift to alkaline pH (pH 8.0).

Up- and down-regulated genes (log_2_ ratio ≥ +/- 1.5) in response to 200 mM CaCl_2_ exposure and to shift to alkaline pH (pH 8.0) for 5, 15, 30, 45 and 60 minutes are annotated. For each time-point in the analysis, the log_2_ fold change in expression relative to the untreated sample is tabulated. The first time-point of significant differential expression and the subsequent time-points at which each gene is differentially expressed is also tabulated.

TABLE S4. List of up-regulated genes (log_2_ ratio ≥ + 1.5) in the K-means clusters identified in response to 200 mM CaCl_2_ exposure as represented in Fig. 3B.

Six clusters were identified for the up-regulated genes (log_2_ ratio ≥ + 1.5) in the microarray analysis following 200 mM CaCl_2_ exposure for 5, 15, 30, 45 and 60 minutes. The lists for these clusters indicate the genes contained in each cluster, plus the respective annotation and the log_2_ fold change in expression relative to the untreated sample.

TABLE S5. List of up-regulated genes (log_2_ ratio ≥ + 1.5) in the K-means clusters identified in response to a shift to alkaline pH (pH 8.0) as represented in Fig. 3C.

Six clusters were identified for the up-regulated genes (log_2_ ratio ≥ + 1.5) in the microarray analysis following a shift to alkaline pH (pH 8.0) for 5, 15, 30, 45 and 60 minutes. The lists for these clusters indicate the genes contained in each cluster, plus the respective annotation and the log_2_ fold change in expression relative to the untreated sample.

TABLE S6. List of down-regulated genes (log_2_ ratio ≥ - 1.5) in the K-means clusters identified in response to 200 mM CaCl_2_ exposure as represented in Fig. 3D.

Six clusters were identified for the down-regulated genes (log_2_ ratio ≥ - 1.5) in the microarray analysis following 200 mM CaCl_2_ exposure for 5, 15, 30, 45 and 60 minutes. The lists for these clusters indicate the genes contained in each cluster, plus the respective annotation and the log_2_ fold change in expression relative to the untreated sample.

TABLE S7. List of down-regulated genes (log_2_ ratio ≥ - 1.5) in the K-means clusters identified in response to a shift to alkaline pH (pH 8.0) as represented in Fig. 3E.

Six clusters were identified for the down-regulated genes (log_2_ ratio ≥ - 1.5) in the microarray analysis following a shift to alkaline pH (pH 8.0) for 5, 15, 30, 45 and 60 minutes. The lists for these clusters indicate the genes contained in each cluster, plus the respective annotation and the log_2_ fold change in expression relative to the untreated sample.

TABLE S8. Primer sequences for RT-qPCR verification of the microarray analysis. The table includes the ORFs selected for verification, the respective gene name, the oligonucleotide sequences and the size of the expected amplicon.
