# Supplementary figures and images for: Mutual independence of alkaline‐ and calcium‐mediated signalling in Aspergillus fumigatus refutes the existence of a conserved druggable signalling nexus
Source: Mol Microbiol. 2017 Nov 14;106(6):861–75. doi: 10.1111/mmi.13840 (PMC5725717; doi:10.1111/mmi.13840)

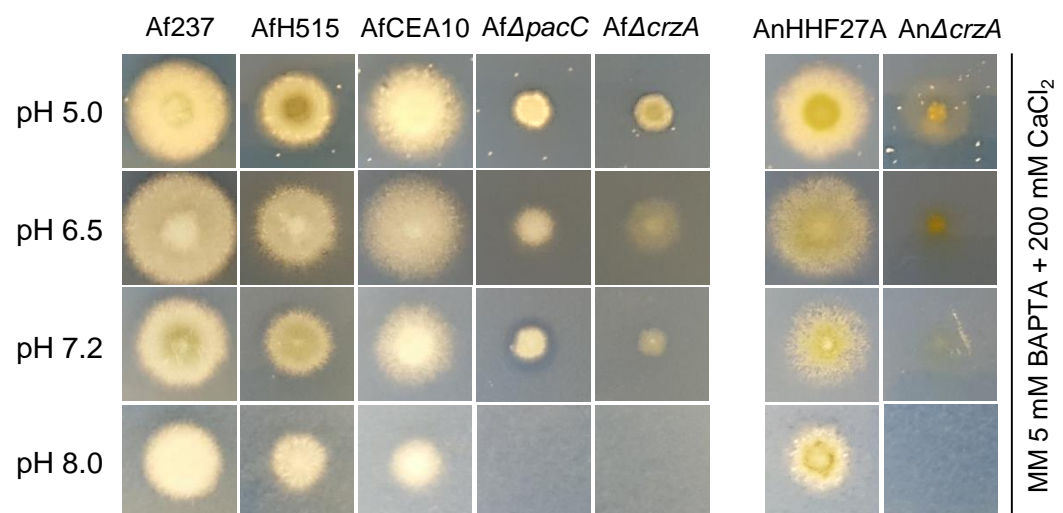

Supplement: Supplementary file 2 — Supporting Figure S1 [file MMI-106-861-s002.pdf]

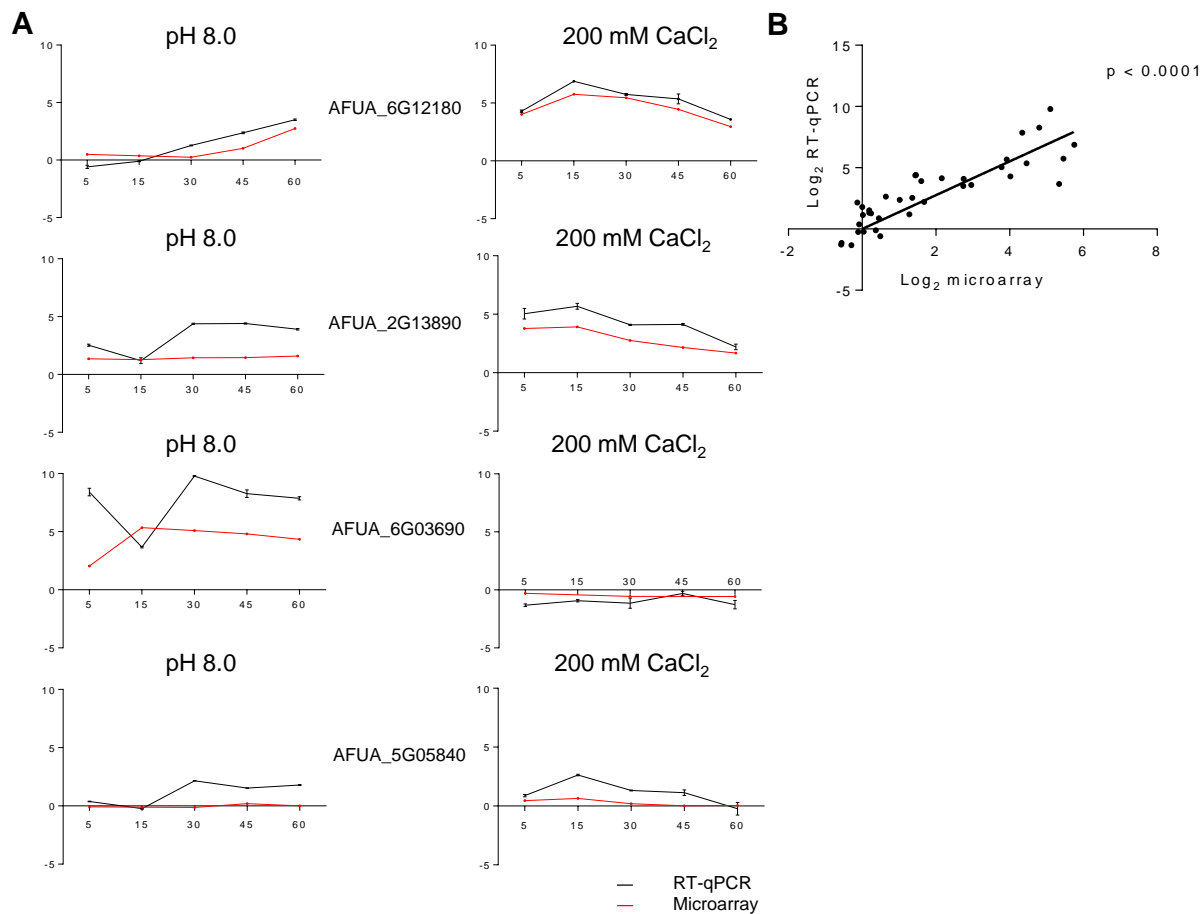

Supplement: Supplementary file 3 — Supporting Figure S2 [file MMI-106-861-s003.pdf]

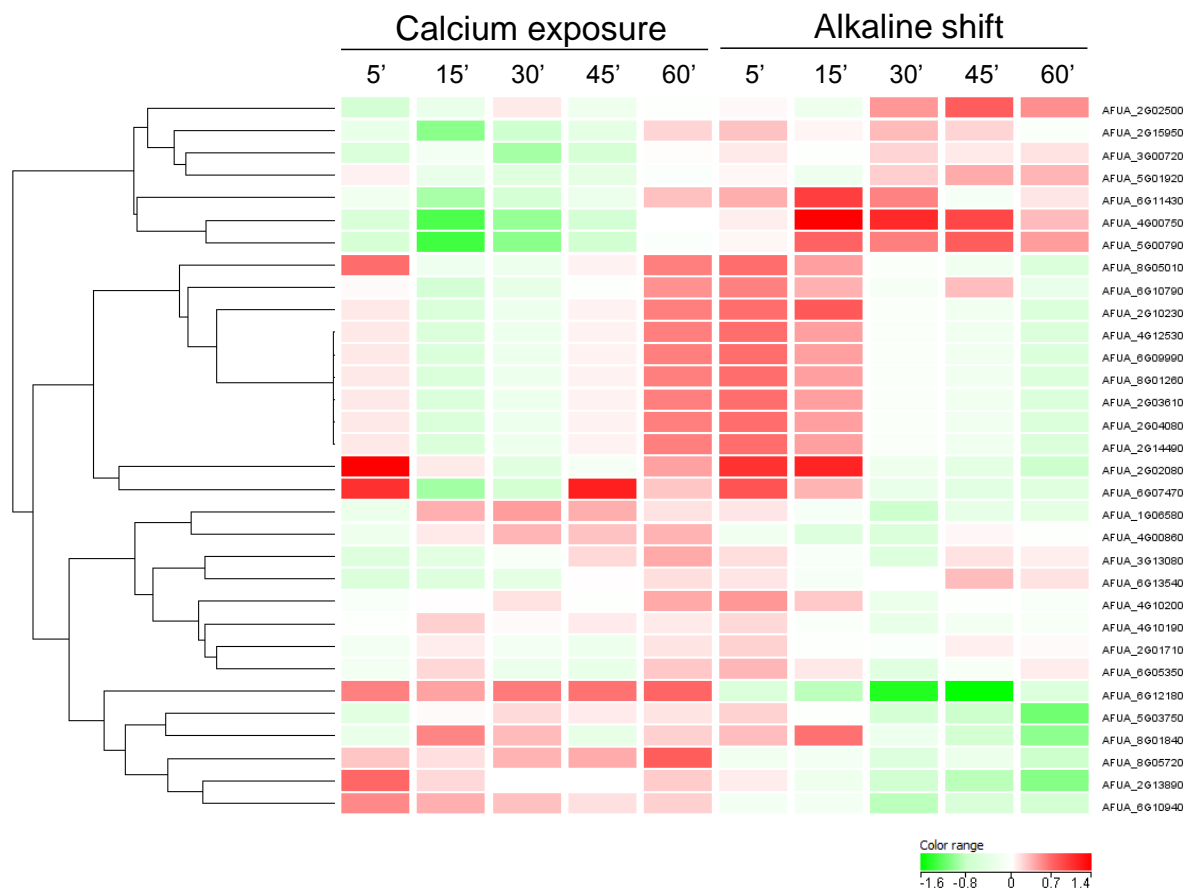

Supplement: Supplementary file 4 — Supporting Figure S3 [file MMI-106-861-s004.pdf]

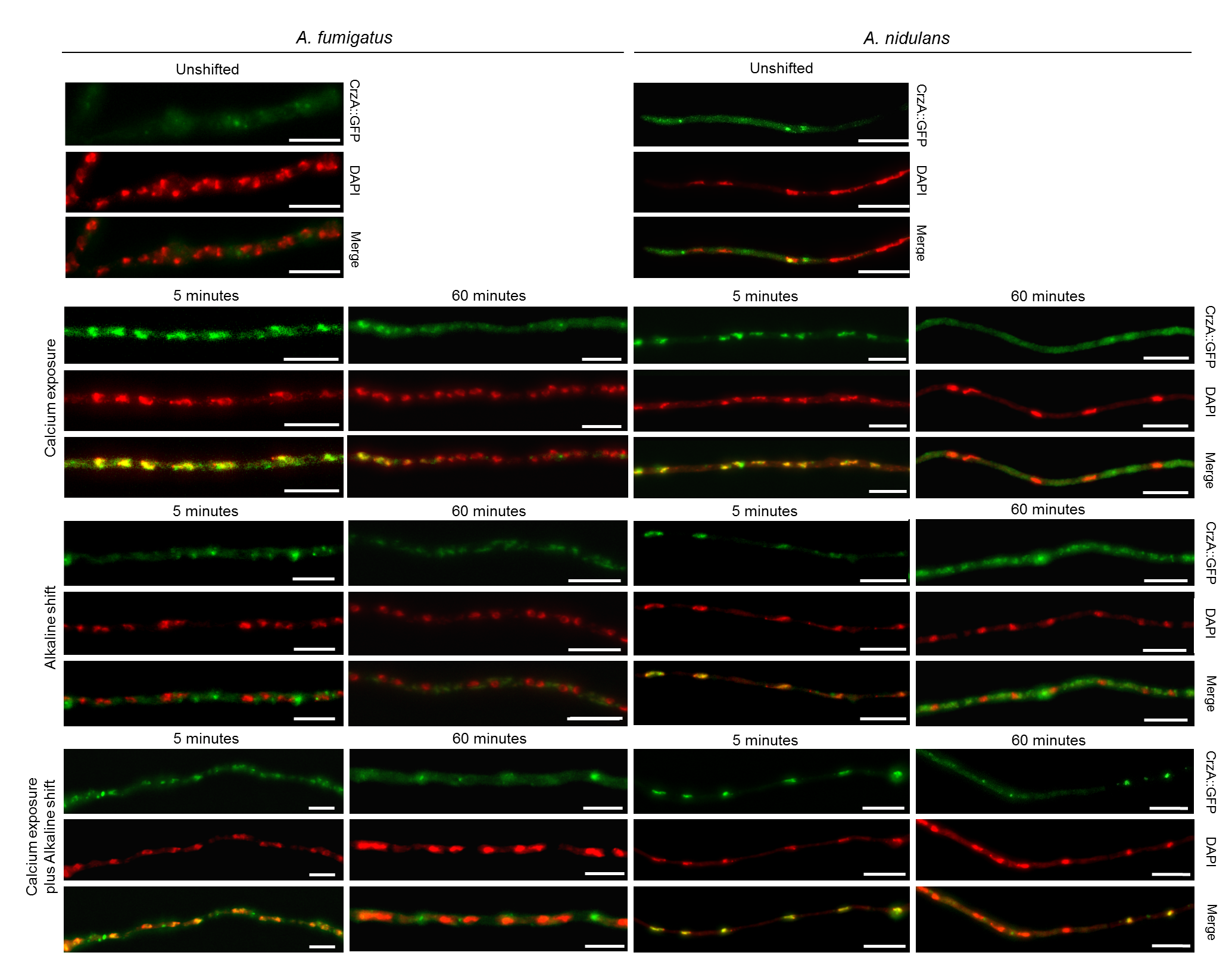

Supplement: Supplementary file 5 — Supporting Figure S4 [file MMI-106-861-s005.tif]
